# Implementing Germ Defence digital behaviour change intervention via all primary care practices in England to reduce respiratory infections during the COVID-19 pandemic: an efficient cluster randomised controlled trial using the OpenSAFELY platform

**DOI:** 10.1186/s13012-023-01321-z

**Published:** 2023-12-04

**Authors:** Ben Ainsworth, Jeremy Horwood, Scott R. Walter, Sascha Miller, Melanie Chalder, Frank De Vocht, James Denison-Day, Martha M. C. Elwenspoek, Helen J. Curtis, Chris Bates, Amir Mehrkar, Seb Bacon, Ben Goldacre, The OpenSAFELY Collaborative, Alex J. Walker, Brian MacKenna, Peter Inglesby, Caroline E. Morton, Jessica Morley, George Hickman, Richard Croker, David Evans, Tom Ward, Nicholas J. DeVito, Louis Fisher, Amelia C. A. Green, Jon Massey, Rebecca M. Smith, William J. Hulme, Simon Davy, Colm D. Andrews, Lisa E. M. Hopcroft, Henry Drysdale, Iain Dillingham, Robin Y. Park, Rose Higgins, Christine Cunningham, Milan Wiedemann, Linda Nab, Steven Maude, Ben F. C. Butler-Cole, Thomas O’Dwyer, Catherine L. Stables, Christopher Wood, Andrew D. Brown, Victoria Speed, Lucy Bridges, Andrea L. Schaffer, Caroline E. Walters, Christopher T. Rentsch, Krishnan Bhaskaran, Anna Schultze, Elizabeth J. Williamson, Helen I. McDonald, Laurie A. Tomlinson, Rosalind M. Eggo, Kevin Wing, Angel Y. S. Wong, John Tazare, Daniel J. Grint, Sinead Langan, Kathryn E. Mansfield, Ian J. Douglas, Stephen J. W. Evans, Liam Smeeth, Jemma L. Walker, Viyaasan Mahalingasivam, Thomas E. Cowling, Emily L. Herrett, Ruth E. Costello, Bang Zheng, Edward P. K. Parker, Rohini Mathur, Harriet Forbes, Jonathan Cockburn, John Parry, Frank Hester, Sam Harper, Pippa Craggs, Richard Amlôt, Nick Francis, Paul Little, John Macleod, Michael Moore, Kate Morton, Cathy Rice, Jonathan Sterne, Beth Stuart, Lauren Towler, Merlin L. Willcox, Lucy Yardley

**Affiliations:** 1 School of Psychology, University of Southampton, Southampton, UK; 2 Department of Psychology, University of Bath, Bath, UK; 3 NIHR Applied Research Collaboration West (NIHR ARC West), University Hospitals Bristol and Weston NHS Foundation Trust, Bristol, UK; 4 Centre for Academic Primary Care (CAPC), Bristol Medical School, Population Health Sciences, University of Bristol, Bristol, UK; 5 NIHR Health Protection Research Unit (HPRU) in Behavioural Science and Evaluation at University of Bristol, Bristol, UK; 6 Bristol Medical School, Population Health Sciences, University of Bristol, Bristol, UK; 7 Nuffield Department of Primary Care Health Sciences, The Bennett Institute for Applied Data Science, University of Oxford, Oxford, UK; 8 TPP, Leeds, UK; 9 Behavioural Science and Insights Unit, UK Health Security Agency, London, UK; 10 Faculty of Medicine, University of Southampton, Southampton, UK; 11 Public Contributor, Southampton, UK; 12 Faculty of Medicine and Dentistry, Wolfson Institute of Population Health, Queen Mary University of London, London, UK; 13 School of Psychological Science, University of Bristol, Bristol, UK

**Keywords:** Respiratory tract infections, Primary care, COVID-19, Behaviour change, Digital medicine, eHealth, Infection control, RCT, Efficient trial design

## Abstract

**Background:**

Germ Defence (www.germdefence.org) is an evidence-based interactive website that promotes behaviour change for infection control within households. To maximise the potential of Germ Defence to effectively reduce the spread of COVID-19, the intervention needed to be implemented at scale rapidly.

**Methods:**

With NHS England approval, we conducted an efficient two-arm (1:1 ratio) cluster randomised controlled trial (RCT) to examine the effectiveness of randomising implementation of Germ Defence via general practitioner (GP) practices across England, UK, compared with usual care to disseminate Germ Defence to patients. GP practices randomised to the intervention arm (*n* = 3292) were emailed and asked to disseminate Germ Defence to all adult patients via mobile phone text, email or social media. Usual care arm GP practices (*n* = 3287) maintained standard management for the 4-month trial period and then asked to share Germ Defence with their adult patients. The primary outcome was the rate of GP presentations for respiratory tract infections (RTI) per patient. Secondary outcomes comprised rates of acute RTIs, confirmed COVID-19 diagnoses and suspected COVID-19 diagnoses, COVID-19 symptoms, gastrointestinal infection diagnoses, antibiotic usage and hospital admissions. The impact of the intervention on outcome rates was assessed using negative binomial regression modelling within the OpenSAFELY platform. The uptake of the intervention by GP practice and by patients was measured via website analytics.

**Results:**

Germ Defence was used 310,731 times. The average website satisfaction score was 7.52 (0–10 not at all to very satisfied, *N* = 9933). There was no evidence of a difference in the rate of RTIs between intervention and control practices (rate ratio (RR) 1.01, 95% *CI* 0.96, 1.06, *p* = 0.70). This was similar to all other eight health outcomes. Patient engagement within intervention arm practices ranged from 0 to 48% of a practice list.

**Conclusions:**

While the RCT did not demonstrate a difference in health outcomes, we demonstrated that rapid large-scale implementation of a digital behavioural intervention is possible and can be evaluated with a novel efficient prospective RCT methodology analysing routinely collected patient data entirely within a trusted research environment.

**Trial registration:**

This trial was registered in the ISRCTN registry (14602359) on 12 August 2020.

**Supplementary Information:**

The online version contains supplementary material available at 10.1186/s13012-023-01321-z.

Contributions to the literature

- Due to the need to rapidly implement the Germ Defence intervention during the COVID-19 pandemic, a novel efficient trial design was adopted, evaluating an active intervention across all GP practices in England.
- The paper outlines the efficient trial design, where no GP practice or patient recruitment was required, and GP practices were not required to send the research team any data, with all outcomes assessed using anonymous patient record data in situ via the OpenSAFELY trusted research environment and website analytics.

## Introduction

The COVID-19 pandemic has highlighted the importance of behavioural strategies for controlling infection transmission. Effective implementation of good hygiene practices as public health measures (including social distancing, self-isolation, handwashing, mask wearing and ventilation) was vital to control the spread until a vaccine was developed [1].

Supporting behaviour change is a complex process that requires an in-depth understanding of why people do/do not engage in target behaviours [2] and tailored support to facilitate engagement. In line with this, there have been calls for major research to develop and evaluate behavioural, environmental, social and systems interventions [3]. Shortly before the H1N1 pandemic, ‘Germ Defence’ was developed to reduce virus transmission within homes. Germ Defence is a digital behaviour change intervention that provides accessible, tailored advice using behaviour change techniques to improve infection control behaviours such as handwashing, social distancing and ventilation in the home.

Reducing within-household transmission pathways is important in contexts where inter-household contact is reduced (i.e. during lockdowns and self-isolation [4, 5]) as well as within more freely mixing circumstances where infectious individuals can expose cohabitants inadvertently to high virus levels leading to increased risk of infection and possibly more severe disease [6]. In a systematic review of digital behavioural interventions to improve hygiene behaviours in the community, we found several studies demonstrating improvement across self-report measures across kindergartens workplaces and restrooms [7]. Only one (‘Germ Defence’) demonstrated improvements using objective measures (reduced consultations and antibiotic prescriptions).

In a previous trial of Germ Defence, 20,066 adults from UK primary care practices were randomised to be given access to Germ Defence reported fewer respiratory tract infections (mean 0.84 vs 1.09 in control group), fewer family member infections and less severe infections [8]; primary consultations and antibiotic prescriptions were also significantly reduced in the intervention group. Rapid co-participatory methods were used to update and optimise Germ Defence for the COVID-19 pandemic https://www.germdefence.org/ [9]. After initial dissemination via clinical and public health networks, social media and press coverage, an analysis of 28,825 users showed an effect size on intentions to improve infection control behaviours similar to that observed in the original trial and recommended wider promotion through primary care and public health networks [10]. It is vital that such digital behaviour change interventions can be implemented effectively, rapidly and at scale. Therefore, we aimed to examine whether the Germ Defence intervention is as follows:

1. Could be disseminated to patients via general practitioner (GP) practices
2. Decreased the number of respiratory tract infection diagnoses in GP practices
3. Decreased other transmissible infections (COVID-19, gastrointestinal)
4. Decreased associated healthcare utilisation (consultations, hospital admissions, antibiotic usage)

Historically, NHS (National Health Service) electronic health record data has been accessed by researchers via a process of pseudonymisation (replacing explicit identifiers such as name and address with a pseudonymous identifier) followed by dissemination of a subset of patients’ records for local analysis. Recently, published UK (United Kingdom) Department of Health and Social Care policy [11] states that detailed electronic health records data should instead be analysed within a secure Trusted Research Environment (TRE). Concerns have been raised that TREs are not suitable for following up patients in an RCT [12].

We used a novel efficient trial design to evaluate the effectiveness of implementing Germ Defence through all GP practices in England being asked to send the intervention to their adult patients. The efficient trial design meant that no GP practice or patient recruitment was required, and GP practices were not required to send the research team any data, with all outcomes assessed using anonymous patient record data in situ via the OpenSAFELY platform trusted research environment and website analytics.

## Method

### Design

As detailed in our protocol [13], this was an efficient pragmatic two-arm (1:1 ratio intervention versus usual care) cluster randomised trial, disseminating Germ Defence to all GP practices in England to reduce respiratory tract infections (RTI). Randomisation was conducted by the independent Bristol Trials Centre (BTC). The 133 NHS Clinical Commission Groups (CCGs: NHS bodies responsible for the planning health care services for their local area) in England were divided into blocks according to region, and equal numbers in each block were randomly allocated to intervention or usual care. The randomisation schedule was generated in Stata statistical software by a BTC statistician not otherwise involved in the enrolment of general practices into the study. The principal investigators, the study statistician and research team remained blinded to the identity of randomised practices until the end of the study.

### Setting and participants

All GP practices in England registered with NHS Digital (*N* = 6579) were included to ensure that the intervention was rolled out across demographically and geographically diverse regions.

### Sample size considerations

To detect a relative risk reduction of 0.14 with 90% power (alpha 0.05), based on the previous Germ Defence implementation, PRIMIT trial [8] was calculated to require 11,124,176 participants from approximately 1484 practices (accounting for clustering). We randomised all GP practices in England, aiming for at least 25% of GP practices of those contacted successfully disseminating the intervention to their patients.

### Intervention

Germ Defence content was rapidly adapted throughout the pandemic using state-of-the-art evidence, theory and the person-based approach [14], in order to ensure the advice remained up to date and appropriate. Content, design and structure were iteratively optimised via co-participatory approaches with the general public in order to ensure the intervention was as accessible, credible and motivating as possible [9]. On the first page participants reached, they could access content in 25 languages as well as infographics (which were also translated into other languages) that they could share with people who were not able to access digital content.

The original Germ Defence intervention drew on the theory of planned behaviour [15] and protection motivation theory [16] to address user motivations and intentions, employing additional theory-based behaviour change techniques such as an if–then planning and self-monitoring to help users implement their handwashing intentions. Drawing on the RE-AIM implementation framework [17], data from the original RCT of the intervention was analysed to examine intervention reach and showed that the intervention was equally effective across gender and age and was particularly effective for people with low and high levels of education [18].

The single-session intervention sought to improve users’ awareness of risks of infection and transmission, increase skills and confidence to reduce risks and use behaviour change techniques (such as making if–then plans) to support behaviours. The Germ Defence content was tailored such that a user selected one of four streams that was relevant to the user’s situation:

1. To protect themselves generally
2. To protect others if the user was showing symptoms
3. To protect themselves if household member(s) showed symptoms
4. To protect a household member who is at high risk

Clear and detailed advice was provided for self-isolating, social distancing, cleaning, wearing face coverings, ventilation and handwashing.

### Intervention implementation

An initial email to practices was drafted by the research team that contained a unique weblink to the Germ Defence website and asked practices to disseminate this to all their adult patients (aged 16 +) via mobile phone text, email or social media. This email was iteratively optimised in pilot interviews with nurses, GPs and administrative staff from six practices to ensure it was acceptable and engaging. Reasons that practices might not engage were discussed (e.g. not enough time, did not perceive benefits, did not typically engage in research, concerns around privacy), and email content was refined to address these barriers.

To further support engagement, the email linked to a trial information website that addressed key concerns and frequently asked questions in more detail [19]. Practices did not need be a research active practice or to sign up to take part in the study; the practice-unique Germ Defence weblink allowed the study team to detect their involvement once patients accessed the Germ Defence intervention. The email also contained suggested text for patient mobile phone message and email. This was also made available in Bengali, French, Polish, Portuguese, Punjabi and Urdu.

On 10 November 2020, intervention arm practices were emailed (see Supplementary file 1) with a practice-specific weblink to the Germ Defence website and asked to disseminate this to all their adult patients (aged 16 +) via mobile phone text, email or social media. Two reminder emails were sent on 25 November and 10 December 2020 to intervention arm practices (see Supplementary file 2).

Data suggest that 16% of the GP practice email addresses forwarded by NHS Digital to the study team did not work, with a total of 613 ‘undelivered’ emails recorded in response to Germ Defence’s initial approach to intervention practices in England. This was usually because registered email addresses were out of date. During the intervention delivery phase, all invalid email addresses were investigated further via a series of manual Internet searches and telephone calls to practices, replacing invalid emails with new information as appropriate. This follow-up effort improved the data quality by around a third.

Patients at GP practices randomised to the usual care arm received standard management for the 4-month (17 weeks) trial period. On 10 March 2021, usual care arm practices were emailed a generic weblink to Germ Defence and asked to disseminate it to all their adult patients.

### Measures and outcomes

The primary outcome was the rate of GP presentations for respiratory tract infections (RTI) per registered patient. Secondary outcomes comprised the rates of acute RTIs, COVID-19 diagnoses, COVID-19 symptoms, gastrointestinal infection diagnoses, antibiotic prescriptions and hospital admissions. COVID-19 symptoms were defined using two different code lists: one designed for high sensitivity and the other for high specificity. Each outcome was defined using SNOMED-CT codelists (see Supplementary file 3 and GitHub repository). A consultation for a specific outcome was identified if a patient had a code from the codelist recorded on a given day. If a patient had multiple codes from the same codelist on the same day, this was counted as one consultation. The number of such consultations divided by the number of patients formed the consultation rate.

All health outcomes were analysed using routinely recorded clinical and patient information in GP practice data. All data were linked, stored and analysed securely within the OpenSAFELY platform, https://opensafely.org/, a trusted research environment (TRE) enabling secure, transparent analysis of electronic health records. Data included pseudonymised fields such as coded diagnoses, medications, physiological parameters, patient age, patient ethnicity and deprivation score of the practice area. No free text data were included. All code used in this study is shared openly for review and re-use under MIT open licence: https://github.com/opensafely/GermDefence. Detailed pseudonymised patient data is potentially re-identifiable and therefore not shared. Primary care records managed by the GP software provider, TPP, were linked to admitted patient care (APC) data through OpenSAFELY. Practice allocations were ingested into the OpenSAFELY platform and linked to pseudonymised practice IDs by TPP and made accessible to the study team by OpenSAFELY.

A further secondary outcome, uptake of the intervention by GP practices, was monitored using embedded code in a unique Germ Defence website link given to each practice. When practices communicated the unique weblink to their patients, the study team were able to record usage of the weblink. Uptake was measured using website analytics such as number of users per practice, average time spent on the Germ Defence website and pages visited, monitored using Matomo to ensure privacy [13, 20]. In line with MRC (Medical Research Council) guidelines for evaluating complex interventions [21], we also sought to understand mechanisms of action by aggregating individual self-report measures of infection control behaviours (social distancing, self-isolation, wearing masks, handwashing, cleaning/disinfecting, ventilation) collected by the Germ Defence website and combined this with metrics of engagement with key intervention behavioural components (e.g. pages viewed, amount of time spent on intervention).

### Patient and public involvement

Patient and public involvement (PPI) feedback was a key part of the co-participatory approach of the development of Germ Defence, in which members of the public were invited to feed back about the website and study in order to optimise and update it. A public contributor (C. R.) was a coinvestigator on the study team and contributed to writing the research proposal, updating and optimising the content of the intervention (including optimising intervention communications sent to patients by practices) and co-authoring the papers. Study materials were also reviewed by PPI representatives from the NIHR (National Institute of Health Research) Clinical Research Network (CRN).

### Data analysis

#### Summary of baseline data

This cluster randomised controlled trial was analysed at the practice level. Randomisation was carried out at practice level, and we did not have direct feedback on whether practices distributed the Germ Defence information to all, some or potentially no patients, nor whether individual patients were offered the information and made use of it. We, therefore, conducted all analyses using aggregated data at the practice level and considered each practice as a unit for the purpose of analysis. Outcome (consultations) and covariate data (median age, proportion of females, proportion from an ethnic minority, deprivation of practice area) from patient-level records were aggregated into weekly practice-level time-series data prior to analysis, covering the period from 17 weeks prior to randomisation until 17 weeks after randomisation (14th July 2020 to 15th March 2021) to achieve a target minimum of 15% infection rate.

Primary care data in the OpenSAFELY system at the time of analysis represented approximately 40% of practices in England [22]. Analyses of health outcomes were applied to 2498 practices.

#### Intention-to-treat analyses

The primary analysis used a standard intention-to-treat approach. For each of the eight health outcomes, rates of consultations per registered patient were compared at practice level between intervention and control groups for the 17-week post-intervention period. This was done using negative binomial regression with the consultation count as the outcome, the number of registered patients as the offset and the binary indicator of intervention/control group as the only independent variable.

#### Controlled interrupted time-series analyses

An additional analysis was performed for the same eight health outcomes using a controlled interrupted time-series (CITS) approach to understand temporal changes related to the intervention as distinct from the time-agnostic intention-to-treat approach. This was implemented within a generalised linear-mixed modelling framework by applying negative binomial regression to weekly level data spanning pre- and post-intervention periods for both the intervention and control groups. Data was also disaggregated by practice, allowing random intercepts at practice level. Variables included in the model were as follows: consecutively numbered weeks to capture a log-linear trend, intervention-control indicator, pre-post-intervention indicator and all two- and three-way interactions between these. Additional covariates included calendar month to capture seasonal effects and practice-level indicators such as area-level deprivation, median patient age and sex distribution represented as the proportion of females.

### Process analysis

#### Implementation process

Germ Defence website usage recorded from the unique identifying website links sent by each practice was used to examine whether intervention engagement (i.e. a practice effectively communicating link to patients) was predicted by practice characteristics (such as indices of deprivation, NHS Quality and Outcomes Frameworks).

#### Individual intervention usage

A range of additional behavioural mechanisms, overall patterns of practice and user engagement were described using website analytics. Analytics included number of users per practice, average time spent on the Germ Defence website and pages visited.

#### Association with health outcomes

To understand the mechanisms of action in the intervention, we examined the association between the rate of website usage within a practice (number of users divided by number of registered patients) and the rate of each health outcome (consultations per registered patient). A negative binomial model was applied to practice level data, and the association of interest was adjusted for decile of deprivation, proportion of patients from an ethnic minority and median age. This was done for all practices and then separately for a subset of practices that had greater than 1% uptake.

### Information governance and ethical approval

NHS England is the data controller for OpenSAFELY-TPP, TPP is the data processor and all study authors using OpenSAFELY have the approval of NHS England. This implementation of OpenSAFELY is hosted within the TPP environment which is accredited to the ISO 27001 information security standard and is NHS IG (information governance) Toolkit compliant [23, 24].

Patient data has been pseudonymised for analysis and linkage using industry standard cryptographic hashing techniques; all pseudonymised datasets transmitted for linkage onto OpenSAFELY are encrypted; access to the platform is via a virtual private network (VPN) connection, restricted to a small group of researchers; the researchers hold contracts with NHS England and only access the platform to initiate database queries and statistical models; all database activity is logged; and only aggregate statistical outputs leave the platform environment following best practice for anonymisation of results such as statistical disclosure control for low cell counts [25].

The OpenSAFELY research platform adheres to the obligations of the UK General Data Protection Regulation (GDPR) and the Data Protection Act 2018. In March 2020, the Secretary of State for Health and Social Care used powers under the UK Health Service (Control of Patient Information) Regulations 2002 (COPI) to require organisations to process confidential patient information for the purposes of protecting public health, providing healthcare services to the public and monitoring and managing the COVID-19 outbreak and incidents of exposure; this sets aside the requirement for patient consent [26]. This was extended in November 2022 for the NHS England OpenSAFELY COVID-19 research platform. In some cases of data sharing, the common law duty of confidence is met using, for example, patient consent or support from the Health Research Authority Confidentiality Advisory Group [27].

Taken together, these provide the legal bases to link patient datasets on the OpenSAFELY platform. GP practices, from which the primary care data are obtained, are required to share relevant health information to support the public health response to the pandemic and have been informed of the OpenSAFELY analytics platform.

This study was approved by the Health Research Authority, Yorkshire & The Humber—Leeds West Research Ethics Committee (Ref.: 20/YH/0261).

## Results

**Fig. 1:**
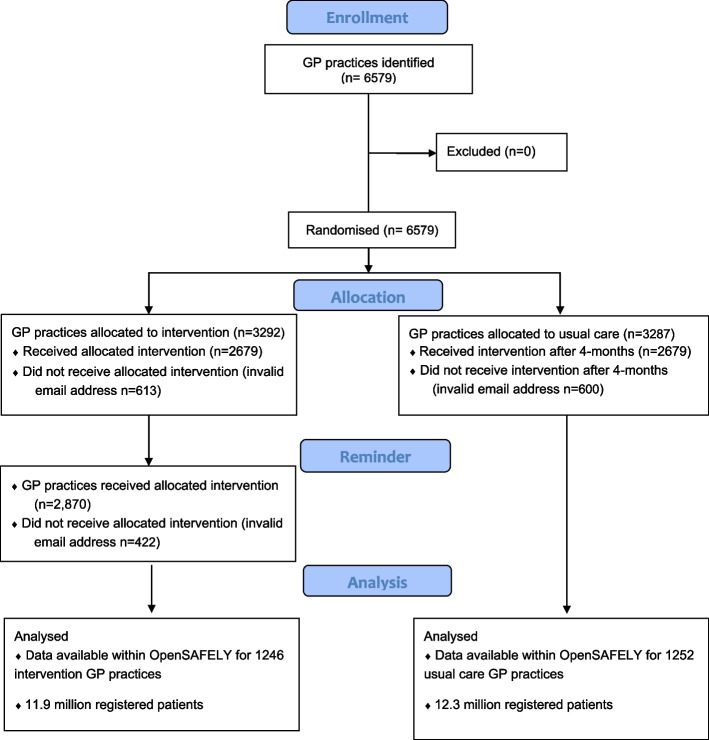
Germ Defence trial CONSORT flow diagram

The initial 10 November 2020 email to 3292 intervention arm practices from 133 CCGs reached 2679 GP practices. The subsequent reminder emails on 25 November and 10 December 2020 reached 2870 GP practices (Fig. 1).

Data show that the Germ Defence website was viewed by patients from 16% of the intervention arm general practices approached as part of the trial. This is based on analysis of website analytics for the usage data which suggest that 10 + clicks were registered for 459 of the 2870 general practices offered in the intervention. A full consort diagram is presented in Fig. 1.

### Intention-to-treat analysis

Using data available within OpenSAFELY, we assessed health outcomes in 1246 intervention practices and 1252 control practices, representing 11.9 million and 12.3 million registered patients, respectively. There was no evidence of a difference in the rate of RTIs (the primary outcome) between intervention and control practices (rate ratio (RR) 1.01, 95% *CI* 0.96, 1.06, *p* = 0.70) (Table 1). This was similarly the case for all other health outcomes, where rate ratios ranged from 0.98 to 1.11 but with no evidence of a difference for any outcome with *p*-values ranging from 0.15 to 0.92.

**Table 1:** Intention-to-treat analysis. Comparison of consultation rates between intervention and control groups for eight health outcomes

	**Control group**	**Intervention group**	**Comparison**	
**Outcome**	Consultations per 1000 registered patients^a^ (95% CI)	Consultations per 1000 registered patients^a^ (95% CI)	Rate ratio (95% CI)	*p*-value
RTIs	14.8 (14.3, 15.3)	15.0 (14.4, 15.5)	1.01 (0.96, 1.06)	0.70
Acute RTIs	14.8 (14.3, 15.3)	14.9 (14.4, 15.4)	1.01 (0.96, 1.06)	0.68
Gastrointestinal infections	0.5 (0.5, 0.5)	0.5 (0.5, 0.6)	1.05 (0.98, 1.13)	0.16
COVID diagnoses	36.9 (36.1, 37.3)	36.8 (36.0, 37.7)	1.00 (0.93, 1.03)	0.92
COVID symptoms (sensitive)	11.5 (10.8, 12.1)	12.4 (11.4, 12.8)	1.05 (0.97, 1.14)	0.22
COVID symptoms (specific)	5.7 (5.1, 6.3)	6.3 (5.6, 7.0)	1.11 (0.96, 1.29)	0.17
Antibiotic use	56.2 (55.2, 57.3)	56.6 (55.5, 57.6)	1.01 (0.98, 1.03)	0.67
Hospital admissions	79.1 (77.8, 80.3)	77.7 (76.5, 78.9)	0.98 (0.96, 1.01)	0.13

^a^Rates represent the 17-week post-intervention period

### Controlled interrupted time series analysis

For all eight health outcomes analysed using CITS models, there was no clear evidence of intervention-related change (Table 2). Although rates did fluctuate over time due to the COVID-infection spikes, seasonal variations and other factors, any such changes affected both intervention and control practices similarly on average. While there was some evidence of an intervention-related trend change for COVID diagnoses (*p* = 0.02), in the context of the many *p*-values in the table, there is considerable potential for this to be a false positive.

**Table 2:** Controlled interrupted time-series estimates assessing intervention-related changes

	**1. Change at time of intervention**		**2. Change in trend following intervention**	
**Outcome**	Rate ratio (95% *CI*)	*p*-value	Rate ratio (95% *CI*)	*p*-value
RTIs	1.02 (1.00, 1.05)	0.10	0.97 (0.93, 1.02)	0.24
Acute RTIs	1.02 (1.00, 1.05)	0.09	0.98 (0.93, 1.02)	0.26
Gastrointestinal infections	1.06 (0.96, 1.17)	0.28	0.95 (0.80, 1.13)	0.57
COVID diagnoses	1.03 (0.99, 1.08)	0.14	1.11 (1.02, 1.20)	0.02
COVID symptoms (sensitive)	1.00 (0.95, 1.05)	0.98	1.04 (0.96, 1.13)	0.32
COVID symptoms (specific)	1.03 (0.94, 1.13)	0.46	0.98 (0.84, 1.13)	0.82
Antibiotic use	1.00 (0.99, 1.01)	0.66	1.01 (0.99, 1.03)	0.22
Hospital admissions	1.00 (0.99, 1.01)	0.62	1.00 (0.98, 1.01)	0.64

Estimates for changes in trend represent relative change in rate over the 17-week post-intervention period compared to the pre-intervention period. Both estimate types adjusted for any background changes in the control group

### Intervention implementation

Details of Germ Defence were emailed to 2870 intervention arm GP practices. The practice unique Germ Defence weblink was accessed by 1094 intervention arm practices (38.1%) at least once. Patient engagement within intervention arm practices ranged from no engagement (when no patient in a practice visited the Germ Defence website) to engagement from 48% of patients registered with the practice. There was no association between practice list size and proportion of uptake (*r* = 0.00, *p* = 0.998). Full details of practice engagement are in Table 3.

**Table 3:** Practice engagement

Usage level	Number of practices	% participating practices	% of total intervention group
None	1777	-	61.9
Low	781	71.5	27.2
Medium	204	18.7	7.1
High	108	9.9	3.8

Usage levels: none 0 user, low 0.01 to 0.99% of list, medium 1.00 to 10.99% of users, high 11 to 100% of users

Further analysis explored whether usage rates [percentage of practice patient list that used the intervention: low (0–0.99%), medium (1–10.99), high (11 +)] differed across varying practice characteristics including population levels of deprivation (IMD), income, employment, education and skills, health and disability, barriers to housing and services, living environment, age, proportion of minority ethnic group and practice quality and outcomes framework performance. Engagement did not differ across any metrics except for ethnicity proportion, in which practices with high levels of uptake (> 11%) had a lower proportion of minority ethnic groups (10.5%) compared to practices with lower levels of use (low: 15.1%, medium: 15.8%). We report analysis of all characteristics in detail in Supplementary file 4.

### Individual intervention usage

The trial intervention link was used 310,731 times, of which 163,991 ‘bounced’, i.e. did not engage beyond first the page. Access to the Germ Defence website using the generic (‘non-trial’) link also increased substantially during the trial period. This is likely due to trial users sharing the website (e.g. with their family or via social media), but these additional users were not included in our analysis. A total of 97.29% (298,752) of users on the trial website were from patients who had been sent a text message from an intervention arm GP practice, with remaining visits via practice social media or GP websites.

Average satisfaction score after using the website was 7.52 (0 meaning not at all satisfied, 10 meaning very satisfied, *N* = 9933). The mean number of page views was 5.2 (standard deviation (SD) 7.2). In ‘engaged’ users who did not bounce, the mean page view was 9.8 (*SD* 8.4), which included all key content targeted at reducing transmission.

While using the intervention, users reported their intentions to improve all infection prevention behaviours (handwashing *d* = 0.48, social distancing *d* = 0.20, ventilation, *d* = 0.32, cleaning/disinfecting *d* = 0.48, wearing face coverings *d* = 0.35, and self-isolation *d* = 0.46) from their current behaviour levels. Further details of current vs. intended behaviours are reported in Supplementary file 4.

### Practice-level usage and health outcomes

There was no clear evidence of an association between website usage rates and health outcomes either among all intervention practices or among those with a user rate greater than 1% (Table 4). While there was modest evidence of higher usage rates among those with COVID symptoms when looking at all practices, this effect direction was reversed for practices where over 1% of patients used the website.

**Table 4:** Process evaluation. Associations between website user rates and consultation rates for intervention practices

	**1. All intervention practices (*n* = 1246)**		**2. Practices with user rate > 1% (*n* = 129)**	
**Outcome**	Rate ratio (95% CI)	*p*-value	Rate ratio (95% *CI*)	*p*-value
RTIs	0.96 (0.82, 1.14)	0.65	1.07 (0.78, 1.48)	0.68
Acute RTIs	0.96 (0.82, 1.14)	0.65	1.07 (0.78, 1.48)	0.68
Gastrointestinal infections	1.09 (0.90, 1.30)	0.37	1.28 (0.88, 1.86)	0.19
COVID diagnoses	0.98 (0.83, 1.16)	0.84	1.02 (0.73, 1.41)	0.92
COVID symptoms (sensitive)	0.98 (0.82, 1.18)	0.86	0.78 (0.52, 1.17)	0.24
COVID symptoms (specific)	1.23 (1.01, 1.50)	0.04	0.67 (0.40, 1.12)	0.13
Antibiotic use	1.00 (0.85, 1.18)	0.99	0.95 (0.69, 1.31)	0.76
Hospital admissions	1.04 (0.88, 1.23)	0.62	1.02 (0.74, 1.40)	0.91

Rate ratios indicate change in consultation rate for every 10% increase in user rate. Results are shown separately for (1) all intervention practices and (2) for a subset where the user rate was greater than 1% of patients in a practice. All estimates were adjusted for median age, deprivation percentile and the proportion of patients from an ethnic minority

### Efficient trial design and intervention implementation

The trial was endorsed by Chris Whitty, the then chief medical officer (CMO) for England, as a national priority project and adopted by the CRN as an urgent public health portfolio study. These endorsements facilitated the novel design by allowing access to email addresses of all GP practices via NHS Digital. However, despite extensive piloting of the process by which practices and patients could be contacted, several practical barriers were encountered: (i) some email addresses were ‘inactive’ due to organisational name changes or practice closure, (ii) practices expected to be contacted by CRNs to take part in research projects rather than directly from study teams and (iii) the intervention practice individualised Germ Defence weblink being perceived as spam by staff and patients, particularly where patients had never previously received a text message from their practice). While these concerns were all identified before the trial began (and addressed via the ‘FAQ’ for patients and clinical staff) [19], some practices may not have had sufficient time to engage with this content due to the operational pressure of the pandemic.

Overall, the study team received 61 enquiries/concerns from primary care staff, patients/members of the public and staff within local Clinical Research Networks during and immediately after the 4-month implementation period (Table 5). The reasons for these ranged from checking that the study and/or the text from practices was legitimate, and the unique Germ Defence weblink was not some kind of scam (e.g. https://www.unknownphone.com/phone/07800007089).

**Table 5:** Reasons for feedback about study intervention or implementation

Reason for feedback	Number of enquiries			
	**Total**	**Patient/public**	**Practice**	**CRN**
Concerns about unauthorised access to/use of personal details	4	4	-	-
Questioned evidence base/advice	2	1	1	-
Weblinks/wording suggesting potential scam	16	5	9	2
Lack of time to engage/participate	3	-	3	-
Direct GP contact with patient suggesting potential scam	4	-	3	1
Concerns about cost/time implications of sending SMS	19	-	19	-
Technical difficulty with weblink	10	-	9	1
Potential ineligibility	3	-	3	-
Total	61	10	47	4

Because the primary aim of the Germ Defence team was to implement Germ Defence as rapidly and widely as possible (based on the previous evidence of effectiveness), extensive implementation was also undertaken outside the defined trial context. For example, the Germ Defence website and the key messages from Germ Defence were publicised on numerous occasions (via national and local radio, TV, online and print media) and were directly linked to from online government advice for Covid infection control [28].

We encountered no material barriers to importing and linking the trial randomisation schedule into a TRE or to evaluating outcomes using patients’ data in situ through the OpenSAFELY platform rather than via data dissemination.

## Discussion

We implemented a novel efficient trial design which was also the first RCT where follow-up was conducted entirely within a TRE. We did not find any consistent overall evidence that the intervention impacted rates of respiratory tract infections or other health outcomes. Similarly, we found no evidence that higher user rates were associated with changes in health outcome rates at a practice level, although there was evidence that more relevant symptoms within a practice were associated with more website uptake.

Although by comparison to many other trial interventions the reach of the Germ Defence intervention was extremely large (it was accessed more than 300,000 times across England during the trial period), we could only confirm that it was accessed by patients from 16% of the general practices approached, below the 25% of GP practices assumed in the sample size considerations. Therefore, it is hard to draw conclusions from our trial data. While we expected that using Germ Defence would improve infection control behaviours and reduce household virus transmission [7], this did not lead to a determinable reduction in health-related outcomes at GP practice level. It is likely that with such a complex behavioural intervention in a rapidly changing contextual environment, determining a signal would require a larger sample of practices that disseminated the intervention effectively to their patients. Our ability to recruit practices was hampered by 16% of the GP practice email addresses forwarded by NHS Digital to the study not working. We recommend NHS England consider how they can better enable efficient, pragmatic trials by having efficient communication channels to contact all GP practices. If email is the preferred communication channel, then it is essential that generic practice email addresses, rather than email addresses of individuals, are used, and that the email list is kept up to date.

However, these findings should not downplay the importance of the efficient trial design that allowed us to conduct a large-scale prospective randomised controlled evaluation of an active behavioural intervention during a pandemic. Our design allowed us to safely recruit GP practices in England during a national pandemic, and we used several novel techniques to minimise practice burden: (i) we recruited practices en masse via email, removing the lengthy process of contacting individual practices (although to avoid overwhelming practices this method should only be used when rapid enrolment is necessary), (ii) we set up intervention access links that were individualised to practices (meaning that practices merely had to use the intervention and we could remotely track their ‘enrolment’), (iii) we used national routinely collected patient record data (accessed through a secure TRE, OpenSAFELY) to analyse electronic health records and (iv) we recorded anonymous digital intervention analytics to understand how many individual patients used the intervention and how they used it.

There was no indication that the use of Germ Defence was impacted by any factors related to deprivation. Generally, this is encouraging news that digital interventions have potential to support healthcare across the socioeconomic spectrum. However, despite substantial effort to ensure accessibility (such as translation, using infographics), we did see that practices with more patients from minority ethnic groups were less likely to have high levels of use. This needs to be examined in more detail to ensure that scalable digital solutions do not lead to digital exclusion for some groups.

### Limitations

This ‘low burden’ design comes with risks. Evaluating an accessible behavioural intervention that can be passed on via multiple communication pathways means we could not prevent people from using the intervention who were not from our randomised intervention group, nor could we control broad contextual factors that may have led to increased contamination (such as the frequent public health communications about Germ Defence during the study period, users sharing the website with other members of the public or interlinked healthcare services) sharing website details with, e.g. practices randomised to the control group). The nature of behavioural interventions means their core behavioural functions can be communicated through means other than the intervention (for example word of mouth) which would have been outside of our randomisation procedure—but in contexts outside of clinical research would be important communication mechanisms. In our trial, it is likely that such contamination could have reduced possible differences between our intervention and control group. Asking people to register to use Germ Defence could have been a method to control for contamination, but this could also be a barrier to using the intervention and reduced its use. Future research should aim to monitor and control contamination; however, this was not possible within our pragmatic trial of a public health intervention during a pandemic.

Despite the unusually rapid set-up and implementation of this study, Germ Defence was rolled out through primary care over 6 months after the start of the COVID-19 pandemic. It is likely that by this point in time, most people who were concerned about infection control in the home may have already obtained all the advice they wanted and needed. Additionally, the lack of lengthy individual practice enrolment processes may have meant that many practices did not have inclination or time to properly engage with the study, given the need to maintain enhanced infection control measures reduced capacity that were increasing pressure on practices during the study period [29]. The combination of delayed implementation of Germ Defence and reduced enrolment of patients may explain why we were unable to provide evidence that disseminating Germ Defence to patients via GP practices improved health outcomes in GP practices. It should further be noted that because of the low uptake, it is likely our intention-to-treat analyses would have under-estimated any effect had there been one.

However, this means our design accurately reflects ‘real-world’ uptake of such interventions outside of usually tightly constrained trial environments. Further research should use implementation frameworks to understand how to further improve rapid adoption and implementation [30] and encourage healthcare providers to recommend digital health interventions to their patients [31].

## Conclusions

In this study we used a novel, efficient prospective randomised controlled trial methodology to examine the rapid implementation and effectiveness of an active digital behavioural intervention across every GP practice in England. The RCT demonstrated that rapid large-scale implementation of a digital behavioural intervention is possible. While the trial did not demonstrate a difference in primary or secondary outcomes between the arms, we showed that it is possible to link intervention usage with individual practice health outcomes and to determine the effects of behavioural intervention engagement with health outcomes. Further work should explore how to improve rapid implementation and how this design can be applied to other types of intervention.

### Supplementary Information

**Additional file 1: Supplementary File 1.** Intervention arm initial intervention email.

** Additional file 2: Supplementary File 2.** Intervention arm reminder intervention email

** Additional file 3: Supplementary File 3.** Codelists.

** Additional file 4: Supplementary File 4.** Practice characteristics categorized by numbers of patients who visited the website and % uptake per practice.

## Data Availability

All analyses involving health outcomes were conducted in the OpenSAFELY research platform, and data cannot be made available outside that platform other than approved aggregated study results. A GitHub repository containing all analysis scripts used in OpenSAFELY for this study can be found here: https://github.com/opensafely/GermDefence.

## Abbreviations

APC: Admitted patient care
BTC: Bristol Trials Centre
CI: Confidence interval
CITS: Controlled interrupted time series
CCGs: Clinical commissioning groups
CMO: Chief medical officer
COPI: Control of Patient Information Regulations
CRN: Clinical Research Network
GDPR: UK General Data Protection Regulation
GP: General practice
IG: Information governance
ISRCTN: International Standard Randomised Controlled Trial Number
MRC: Medical Research Council
NIHR: National Institute for Health Research
NHS: Nation Health Service
PPI: Patient and public involvement
RCT: Randomised controlled trial
RR: Rate ratio
RTI: Respiratory tract infections
SD: Standard deviation
TRE: Trusted research environment
UK: United Kingdom
VPN: Virtual private network
