# Supplementary material for: Implementing Germ Defence digital behaviour change intervention via all primary care practices in England to reduce respiratory infections during the COVID-19 pandemic: an efficient cluster randomised controlled trial using the OpenSAFELY platform
Source: Implement Sci. 2023 Dec 4;18:67. doi: 10.1186/s13012-023-01321-z (PMC10694966; doi:10.1186/s13012-023-01321-z)
Supplement: Supplementary file 1 — Additional file 1: Supplementary File 1. Intervention arm initial intervention email. [file 13012_2023_1321_MOESM1_ESM.docx]

**Supplementary File 1 – Intervention arm initial intervention email**

Dear colleagues at XXX Practice­

 We have been funded by the National Institute for Health Research (NIHR) and UK Research and Innovation (UKRI) to update the **Germ Defence** **website with COVID-19** **information** and ask general practices across England to promote it to their patients to help prevent a wave of COVID-19 and ‘flu this Winter.

**Chris Whitty, the Chief Medical Officer (CMO) for England, has endorsed this as a national priority project and it has been adopted on to the portfolio of the Clinical Research Networks (CRNs) as an Urgent Public Health Research (UPHR) study. The Royal College of General Practice (RCGP) also supports this work and has recommended that all practices take part.**

**All you need to do is pass this unique link on to your patients aged 16 and over**

 www.germdefence.org/index.html?src=123TEST

 The link (which has been created specifically for your practice) can be sent using the templates provided at the bottom of this e-mail. Texting is the best method of promoting the link, but an example e-mail is provided too. You can also share these messages (with the unique link) on your practice website or via social media.

 Germ Defence is an evidence-based behaviour change website that provides advice on infection control at home. A clinical trial of 20,000 people during the time of the H1N1 pandemic and seasonal ‘flu (published in [The Lancet](https://www.thelancet.com/journals/lancet/article/PIIS0140-6736(15)60127-1/fulltext)) showed that using Germ Defence successfully reduced the number and severity of infections in the home, as well as the need for primary care consultations and antibiotics.

 We are now examining whether the COVID-19 version of Germ Defence will be effective at reducing infections and GP consultations this Winter. Half of practices in England are being asked to promote Germ Defence to their patients now and the other half after Christmas. We will then use anonymised national patient record data to assess the website’s effectiveness.

 The COVID-19 version of Germ Defence was developed by clinicians and scientists at the Universities of Bristol, Southampton and Bath in collaboration with Public Health England. It was co-designed with members of the public to be simple and quick to use, including by people with limited reading or computer skills. The website has already had over 100,000 users, who gave it a median rating of 8/10 for usefulness, and it can be accessed in 20+ languages. GP practices that have already shared the link have not received any patient enquiries about the website.

**Remember, all your practice needs to do is pass this unique link on to your patients**

[www.germdefence.org/index.html?src=123TEST](http://www.germdefence.org/index.html?src=123TEST)

**You do not need to sign up to the study, register to use the Germ Defence website or provide us with any data.**We will record your practice’s participation by monitoring use of the unique link that is sent to patients, allocating one recruit to each practice that promotes the website. There is no financial reimbursement for taking part.

 You can find more information about the study, including frequently asked questions (FAQs) [here](http://www.bristol.ac.uk/primaryhealthcare/researchthemes/roll-out-of-germ-defence-website/).

 Many thanks

 Professor Lucy Yardley, Dr Jeremy Horwood, Professor John Macleod

*Centre for Academic Primary Care, University of Bristol*

**Templates to use when sending the Germ Defence link**

| *Example of a mass text message that can be sent (using MJog, accuRx, iPLATO or similar system) to your patients. If you choose to edit this text, please keep it shorter than 160 characters.*   This website www.germdefence.org/index.html?src=123TEST has scientifically proven advice to reduce COVID-19 infections. It takes 10 minutes – try it out! |
| --- |

| *Example of an e-mail that can be sent to your patients*   We are letting you know about a very useful website called **Germ Defence** which was created by a team of doctors and scientists to give you **advice that has been proven to reduce the spread of viruses in the home**. It can help you plan how to **protect yourself and members of your family**from infection by COVID-19 and ‘flu.  **It’s easy to use and only takes 10 minutes - just click on this link**   www.germdefence.org/index.html?src=123TEST   (If this link does not open when you click on it, just copy and paste it into your web browser)  **Please pass details of the Germ Defence website to your friends and family.**There’s a button at the bottom of the Germ Defence website for sharing by social media.  **If you’d like to know more:**   - Over 20,000 people previously took part in research about Germ Defence - People who followed the advice in Germ Defence had fewer and less severe illnesses - and so did the people they lived with - Results of the study were published in The Lancet medical journal - Germ Defence has been updated with COVID-19 advice to help prevent a wave of COVID-19 and ‘flu this Autumn/Winter - Information about how the Germ Defence website is being evaluated is available [here](http://www.bristol.ac.uk/primaryhealthcare/researchthemes/roll-out-of-germ-defence-website/). |
| --- |
