# Supplementary material for: Implementing Germ Defence digital behaviour change intervention via all primary care practices in England to reduce respiratory infections during the COVID-19 pandemic: an efficient cluster randomised controlled trial using the OpenSAFELY platform
Source: Implement Sci. 2023 Dec 4;18:67. doi: 10.1186/s13012-023-01321-z (PMC10694966; doi:10.1186/s13012-023-01321-z)
Supplement: Supplementary file 2 — Additional file 2: Supplementary File 2. Intervention arm reminder intervention email [file 13012_2023_1321_MOESM2_ESM.docx]

**Supplementary File 2 – Intervention arm reminder intervention email**

Dear colleagues at XXX

The **Germ Defence** **website**gives scientifically proven, practical advice on how your patients can reduce the spread of coronavirus in the home.  Half of general practices in England are being asked to promote it to their patients now and the other half after Christmas.

Chris Whitty, the Chief Medical Officer (CMO) for England, has endorsed this project as a national Urgent Public Health Research (UPHR) priority and it is supported by the Royal College of General Practice (RCGP) who recommended that all practices take part.

**We are e-mailing to remind you that your practice has been randomised to promote Germ Defence immediately.  All you need to do is pass this unique link on to your patients aged 16 and over:** [www.germdefence.org/index.html?src=A86027](http://www.germdefence.org/index.html?src=A86027)

 (If this link does not open when you click on it, please copy and paste it into your web browser.)

This weblink has been specifically generated for your practice, and the numbers at the end of it will allow us to know how many of your patients visited the website. This makes the project as efficient as possible and means that you don’t need to sign up or send us any other information.  So far, more than a hundred practices have sent the weblink out via text message and almost 50,000 patients have already used the Germ Defence website.

The COVID-19 version of Germ Defence was developed by clinicians and scientists at the Universities of Bristol, Southampton and Bath in collaboration with Public Health England. A clinical trial of 20,000 people during the swine ‘flu pandemic showed than an earlier version of Germ Defence successfully reduced respiratory infection frequency and severity as well as the number of GP consultation.  The results were published in [The Lancet](https://www.thelancet.com/journals/lancet/article/PIIS0140-6736(15)60127-1/fulltext).

If you have any questions about the project, please visit our [website](http://www.bristol.ac.uk/primaryhealthcare/researchthemes/roll-out-of-germ-defence-website/) and read the ‘frequently asked questions (FAQs).  If you have already sent out the weblink to your patients, thank you very much – we appreciate your support.

Many thanks

Professor Lucy Yardley, Dr Jeremy Horwood, Professor John Macleod

*Centre for Academic Primary Care, University of Bristol*

**Templates to use when sending the Germ Defence link**

| *Example of a mass text message that can be sent (using MJog, accuRx, iPLATO or similar system) to your patients. If you choose to edit this text, please keep it shorter than 160 characters.*  This website [www.germdefence.org/index.html?src=A86027](http://www.germdefence.org/index.html?src=A86027) has scientifically proven advice to reduce COVID-19 infections. It takes 10 minutes – try it out! |
| --- |

| *Example of an e-mail that can be sent to your patients*   We are letting you know about a very useful website called **Germ Defence** which was created by a team of doctors and scientists to give you **advice that has been proven to reduce the spread of viruses in the home**. It can help you plan how to **protect yourself and members of your family**from infection by COVID-19 and ‘flu.  **It’s easy to use and only takes 10 minutes - just click on this link:**  [www.germdefence.org/index.html?src=A86027](http://www.germdefence.org/index.html?src=A86027)   (If this link does not open when you click on it, please copy and paste it into your web browser.)  **Please pass details of the Germ Defence website to your friends and family.**  There’s a button at the bottom of the Germ Defence website for sharing by social media.   If you’d like to know more:   - Over 20,000 people previously took part in research about Germ Defence - People who followed the advice in Germ Defence had fewer and less severe illnesses - and so did the people they lived with - Results of the study were published in The Lancet medical journal - Germ Defence has been updated with COVID-19 advice to help prevent a wave of COVID-19 and ‘flu this Autumn/Winter - Information about how the Germ Defence website is being evaluated is available [here](http://www.bristol.ac.uk/primaryhealthcare/researchthemes/roll-out-of-germ-defence-website/). |
| --- |
