# Supplementary material for: Implementing Germ Defence digital behaviour change intervention via all primary care practices in England to reduce respiratory infections during the COVID-19 pandemic: an efficient cluster randomised controlled trial using the OpenSAFELY platform
Source: Implement Sci. 2023 Dec 4;18:67. doi: 10.1186/s13012-023-01321-z (PMC10694966; doi:10.1186/s13012-023-01321-z)
Supplement: Supplementary file 3 — Additional file 3: Supplementary File 3. Codelists. [file 13012_2023_1321_MOESM3_ESM.docx]

**Supplementary File 3: Codelists**

Respiratory tract infections

<https://www.opencodelists.org/codelist/user/S_Walter/respiratory-tract-infections/23f7396b/>

Acute respiratory tract infections

<https://www.opencodelists.org/codelist/user/S_Walter/acute-respiratory-tract-infections/75b06bce/>

Gastrointestinal infections

<https://www.opencodelists.org/codelist/user/S_Walter/gastrointestinal-infections/27cf5239/>

COVID-19 diagnoses

<https://www.opencodelists.org/codelist/user/S_Walter/covid-19-diagnoses/310ceaf3/>

COVID-19 symptoms – sensitive

<https://www.opencodelists.org/codelist/user/S_Walter/covid-19-symptoms-sensitive/0fe537dc/>

COVID-19 symptoms – specific

<https://www.opencodelists.org/codelist/user/S_Walter/covid-19-symptoms-specific/28f4ab11/>

Antibiotic prescriptions

[[https://www.opencodelists.org/codelist/user/](https://www.opencodelists.org/codelist/user/jon_massey/germdefence-test-dmd/3f100acc)](https://www.opencodelists.org/codelist/user/)[jon_massey/germdefence-test-dmd/3f100acc](https://www.opencodelists.org/codelist/user/jon_massey/germdefence-test-dmd/3f100acc)
