# Supplementary material for: Implementing Germ Defence digital behaviour change intervention via all primary care practices in England to reduce respiratory infections during the COVID-19 pandemic: an efficient cluster randomised controlled trial using the OpenSAFELY platform
Source: Implement Sci. 2023 Dec 4;18:67. doi: 10.1186/s13012-023-01321-z (PMC10694966; doi:10.1186/s13012-023-01321-z)
Supplement: Supplementary file 4 — Additional file 4: Supplementary File 4. Practice characteristics categorized by numbers of patients who visited the website and % uptake per practice. [file 13012_2023_1321_MOESM4_ESM.docx]

**Supplementary File 4 - Practice characteristics categorized by numbers of patients who visited the website and % uptake per practice**


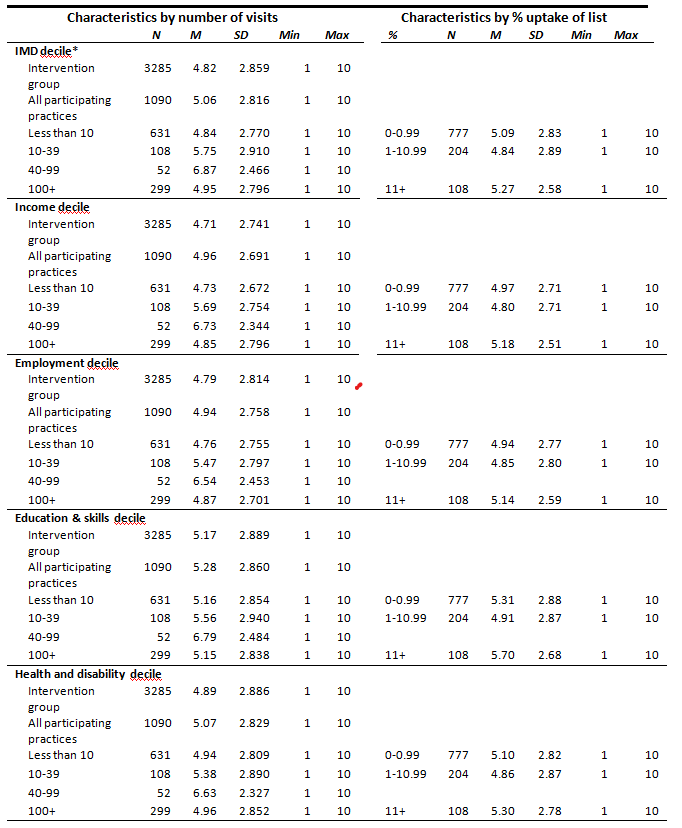


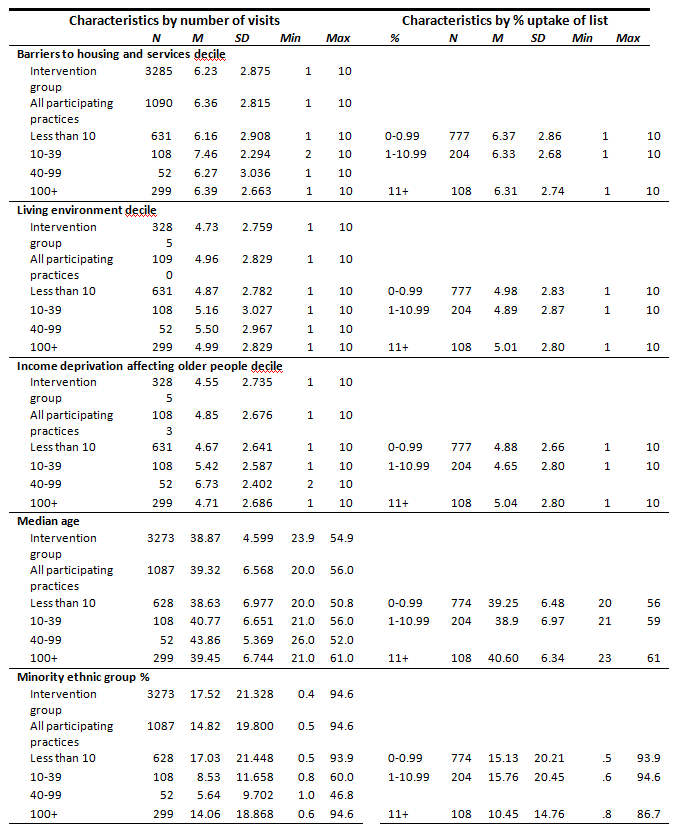


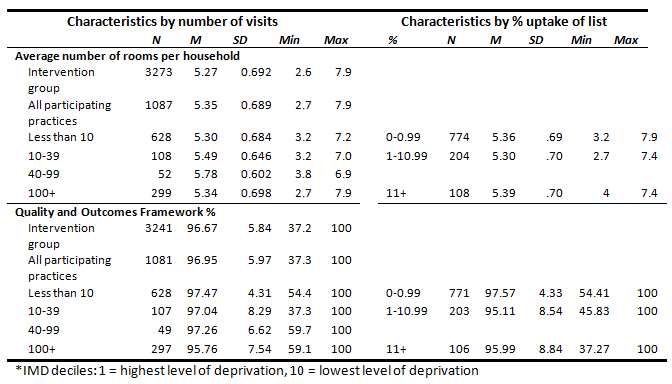


**Self-reported infection control behaviours and intended behaviours in users of Germ Defence**

| **Behaviour**  **(N 12049)** | Current Behaviour  M (SD)  N 10852 | Intended Behaviour  M (SD)  N 12,981 | Mean difference (95% CI) | Paired comparison  (t-test) |
| --- | --- | --- | --- | --- |
| Handwashing | 3.96 (1.10) | 4.29 (0.97) | 0.34 (0.31 to 0.36) | *t*(2817) = 25.7, *p* < .001, d= .48 |
| Self-isolation | 2.93 (1.26) | 3.04 (1.24) | 0.10 (0.09 to 0.11) | t(11967)=21.4, p<.001; d=.20 |
| Social Distancing | 2.59 (1.21) | 2.79 (1.24) | 0.20 (0.19 to 0.21) | t(10852)=33.3, p<.001; d=.32 |
| Ventilation | 2.09 (1.36) | 2.56 (1.48) | 0.48 (0.46 to 0.50) | (t(11361)=51.6, p<.001; d=.48. |
| Face-covering | 2.32 (1.48) | 2.60 (1.51) | 0.28 (0.26 to 0.29) | t(10747)=36.6, p<.001; d=.35 |
| Cleaning / Disinfecting | 3.11 (1.16) | 3.43 (1.15) | 0.32 (0.31 to 0.33) | t(11895)=50.2, p<.001; d=.46 |

**Note**: Self-report scores were collected from website users during periods between 18/9/20 – 4/12/20 and 15/2/21-12/3/21. Data is reported from users who completed measures of current and intended behaviour only.
